# Gender Differences in Throwing Revisited: Sensorimotor Coordination in a Virtual Ball Aiming Task

**DOI:** 10.3389/fnhum.2019.00231

**Published:** 2019-07-18

**Authors:** Dena Crozier, Zhaoran Zhang, Se-Woong Park, Dagmar Sternad

**Affiliations:** ^1^Department of Physics, Northeastern University, Boston, MA, United States; ^2^Department of Biology, Northeastern University, Boston, MA, United States; ^3^Department of Bioengineering, Northeastern University, Boston, MA, United States; ^4^Department of Electrical and Computer Engineering, Northeastern University, Boston, MA, United States

**Keywords:** gender difference, throwing, age-dependency, timing ability, intrinsic rhythmicity

## Abstract

Numerous studies have demonstrated that boys throw balls faster, farther and more accurately than girls. This may be largely due to well-known anatomical and muscle-physiological differences that play a central role in overarm throwing. With the objective to understand the potential contribution of the equally essential coordinative aspects in throwing for this gender difference, this large cross-sectional study examined a simplified forearm throw that eliminated the requirements that give males an advantage.While the overall performance error indeed became similar in the age groups younger than 20 years and older than 50 years, it was attenuated for middle-aged individuals. The gender differences remained in individuals who reported no throwing experience, but females with throwing experience reached similar performance as males. Two fine-grained spatiotemporal metrics displayed similar age-dependent gender disparities: while overall, males showed better spatiotemporal coordination of the ball release, age group comparisons specified that it was particularly middle-aged females that made more timing errors and did not develop a noise-tolerant strategy as males did. As throwing experience did not explain this age-dependency, the results are discussed in the context of spatial abilities and video game experience, both more pronounced in males. In contrast, a measure of rhythmicity developed over successive throws only revealed weak gender differences, speaking to the fundamental tendency in humans to fall into rhythmic patterns. Only the youngest individuals between 5 and 9 years of age showed significantly less rhythmicity in their performance. This computational study was performed in a large cohort in the context of an outreach activity, demonstrating that robust quantitative measures can also be obtained in less controlled environments. The findings also alert that motor neuroscience may need to pay more attention to gender differences.

## Introduction

Gender differences have been the mainstay in the developmental literature on motor, sensory, and cognitive abilities in children from early childhood to adolescence and early adulthood. In the numerous studies that have assessed motor development in various population segments using standardized test batteries, one of the most robust findings is that boys are better in throwing than girls (Thomas and French, 1985; Barnett et al., 2010; Gromeier et al., 2017). Given the evolutionary division of labor into male hunters and female gatherers and the persistent gender stereotypies in education, this finding has invariably raised the issue of nature vs. nurture (Lombardo and Deaner, 2018a,b). In contrast to this literature, studies in motor neuroscience have largely side-stepped the question of sex differences and it has been regarded good practice to average over both equally represented genders. Only recently, the National Institutes of Health have called for attention to sex as a biological variable (Pardue and Wizemann, 2001; McCarthy et al., 2012; Clayton and Collins, 2014; Ritz et al., 2014). The present study examined an experimentally simplified throwing task to further probe into the coordinative differences between males and females using quantitative motor neuroscience methods.

A host of studies have reported differential development between genders in motor skill proficiency from early childhood throughout adolescence. In childhood, boys are generally more proficient than girls in object manipulation, such as in throwing, catching or kicking a ball (Raudsepp and Pääsuke, 1995; van Beurden et al., 2002; Runion et al., 2003; Booth et al., 2004; Ehl et al., 2005). A more recent study by Barnett et al. (2010) reported that while locomotor skills did not show gender differences, boys were significantly more proficient in object control skills than girls, as exemplified in overhand throwing. This disparity widens over time, especially with the onset of puberty (Cratty, 1979; Keogh and Sugden, 1985; Thomas and French, 1985; Burton and Rogerson, 2003). McKay et al. (2017) reported that after age 10, males performed better in gross motor skills, while females performed better in fine motor function (see also Nicholson and Kimura, 1996). Tests of aiming at moving or stationary targets appear to favor males (Watson and Kimura, 1991; but see also Auyeung et al., 2012). Overall, it is fair to say that the literature on perceptual-motor skills is rather mixed. One reason for this discrepancy in the literature is that many tests only measure outcomes and not finer-grained performance features.

Even though a similar rate of girls and boys improved from childhood to adolescence, the girls were unable to catch up with the boys’ performance unless they were also trained at an early age. A meta-analysis of 64 studies on a large set of sensorimotor skills by Thomas and French (1985) reported that specifically throwing differed significantly from other skills: boys outperformed girls by 1.5 standard deviations as early as 4–7 years, and by 12 years, boys outperformed girls by over 3.5 standard deviations. These results suggest that differences in throwing ability were unlikely to be completely rooted in nurture or environmental causes. A disparity between male and females have not only been reported in novices but also in adult athletes (Gromeier et al., 2017).

When reviewing this literature on throwing it is important to mention that this complex coordinative skill can have different goals: throwing may aim to achieve maximum speed or distance or may aim to hit a target with high accuracy; strength requirements depend on the distance to the target. These actions can be performed with the archetypal overarm action, but also with underarm throws, involving wrist flicks and other means of propelling the ball. The type and size of ball also influences the action. The developmental studies largely focused on overarm throwing, which requires specific shoulder mobility and muscle strength enabling the characteristic whip-like torso-shoulder-elbow-wrist coordination. It is not surprising that adolescent boys have an advantage over girls due to their larger and more muscular physiques including several features that favor the arm extension necessary for the whip-like overarm throwing motion, such as shoulder mobility, arm length and muscle strength (Thomas and French, 1985; Lombardo and Deaner, 2018a,b). But the same studies also speculate that this anatomical advantage may not be the only factor for the disparity.

Aside from strength, throwing a projectile also requires fine-tuned arm-hand coordination with a well-controlled timing of the ball release. For example, when throwing a javelin, the whip-like whole-body action only results in the desired distance if the javelin is released at the right moment to translate the momentum into velocity of the javelin at release. When aiming a ball to a target, the demands on the timing of ball release are even more critical: the complex throwing action needs to be oriented to a target in the extrinsic space, i.e., the inter-joint coordination has to be translated from an egocentric to an allo-centric reference frame. A recent study showed how the coordinative solutions are critically dependent on the location of the target, necessitating significantly different strategies (Zhang et al., 2018). We argue that to gain more insights into the observed differences between genders it is useful to tease apart the complex task of throwing into its essential elements. Our study focused on the fundamental sensorimotor features of throwing a ball to a target and asked the question whether this sensorimotor coordination is similarly more advanced in males.

To examine whether this core element of throwing contributes to the gender differences, this study used a throwing task performed in a virtual environment that was reduced to a forearm movement in the horizontal plane with a virtual ball release to hit a target in a virtual work space. This task eliminated the throwing-specific whole-arm coordination with strength and speed requirements and made the spatiotemporal requirements of ball release the central element. Such reduction of complexity and experimental control differs from the developmental approaches but is typical for neuroscientific studies. In previous research, we highlighted that even this controlled throwing task still affords different strategies that optimize the ball release to achieve a precise and accurate target hit (Müller and Sternad, 2004b; Cohen and Sternad, 2009, 2012; Zhang et al., 2018). With this focus on coordinative rather than physical abilities, we hypothesized that females were no longer disadvantaged and that skill should be equally present in both genders (*Hypothesis 1*).

However, the sensorimotor skill of accurate targeting comprises a variety of aspects that have previously led to different conclusions on potential gender disparities. Some studies pointed out that accurate targeting relies heavily on spatial analysis, including spatial orientation and estimating relative distance and velocity (Kimura and Hampson, 1994; Geary, 1995; Wong, 2017). A meta-analysis by Voyer et al. (1995) concluded that males had an advantage in spatial perception and mental rotation, even though the latter has been disputed (Moè, 2009; Lippa et al., 2010). A more recent review of cognitive abilities also suggested a male advantage in some cognitive tasks from early childhood onwards (Miller and Halpern, 2014). Judging distances in space and projecting a trajectory with respect to a target requires cognitive processes where males have performed better (Uttal et al., 2013; Wong, 2017). Based on these results, we hypothesized that male participants would retain their upper hand in the virtual throwing task (*alternative* *Hypothesis*).

In contrast, an extensive literature on timing abilities has failed to arrive at reliable disparities, partly because timing is inherent in a large variety of tasks. For example, studies on extrinsic time estimation have revealed gender differences (McLeod and Ross, 1983; Schiff and Oldak, 1990; Sanders and Sinclair, 2011). When young adults were asked to estimate time to collision based on the optic flow, males gave significantly more accurate estimates (McLeod and Ross, 1983; Schiff and Oldak, 1990). The same disparity was replicated in a later study on spatiotemporal judgments in a virtual reality game (Sanders and Sinclair, 2011). Tests on the ability to internally estimate a specific time interval have also shown that males are more accurate than females (Hancock and Rausch, 2010; Hancock and Block, 2016). On the other hand, when asked to synchronize to a metronome and maintain that extrinsic rhythm after metronome termination, there was no difference between genders (Groves, 1969; Smoll, 1974; Thomas and Moon, 1976; Derri et al., 2001; Pollatou et al., 2005), although some studies revealed that girls performed better than boys when testing synchronization with different limbs (Moore, 1974; Gilbert, 1980; Schleuter and Schleuter, 1985; Flohr, 1991). Following these heterogeneous results, we do not expect differences in rhythmic timing (*Hypothesis 2*).

The present study examined whether a simple target-oriented throwing task that does not require physical strength and speed revealed gender differences across the lifespan. This virtual throwing game had several benefits. First, the virtual rendering implemented a physical model of the task that could provide a reference for the analysis of subjects’ performance. Based on this model, the successful solutions could be derived and precise measures could be extracted that quantified different strategies. Second, the throwing movement only involved a forearm extension without little inter-joint coordination. This experimental reduction eliminated strength and musculo-skeletal factors which are known to give males a significant advantage. Third, the set-up was low-cost and portable and could be used outside the lab to involve a large spectrum of participants. Specifically, this set-up was deployed as a research exhibit in the Museum of Science 1 day per week over 8 months. A group of experimenters invited museum patrons to participate and, in turn, educated them about ongoing research. We tested a cohort of over 400 individuals between 5–65 years of age to investigate how age and gender affected the throwing skill.

## Materials and Methods

### Participants

All subjects in this experiment were visitors at the Museum of Science in Boston, MA, USA. The data were collected from a total of 417 subjects (210 females, 207 males, ages 5–65 years) between October 2015 and May 2016 as part of an outreach activity. According to museum policy, participants had to be at least 5 years old; there was no upper age limit, but fewer older subjects volunteered for the study. Any targeted recruitment of age or gender or any exclusion was not permitted per museum policy. Thirty-two subjects voluntarily disclosed a condition that might have affected their performance (e.g., neurological conditions or shoulder/elbow injuries); these subjects were allowed to participate in the experiment, however, their data were not analyzed. Thus, 385 subjects (191 females, 194 males, ages 5–65 years) were included in the reported results. All methods were approved by the Institutional Review Boards of Northeastern University and the Museum of Science, Boston, MA, USA.

Each data collection required three experimenters to be present, one for recruiting, one for obtaining informed consent and educational information, and one for data collection. A total of nine experimenters, comprising six undergraduate students, two graduate students, and one postdoctoral fellow rotated in the data collection responsibilities each week. All experimenters were trained to follow the same protocol and to give the same verbal instructions to the participants. Subjects or caregivers for young participants gave informed consent prior to participation and completed a one-page survey that collected demographic information and handedness. To assess their previous throwing experience, one prompt on the questionnaire was, “Do you have any experience playing sports that involves throwing (baseball, football, basketball, etc.)? If yes, please specify the activity and how many years of experience you have.” As this data collection was performed in a public place, there were more limitations on the questions than in a laboratory study.

### Demographics

**Figure 1:**
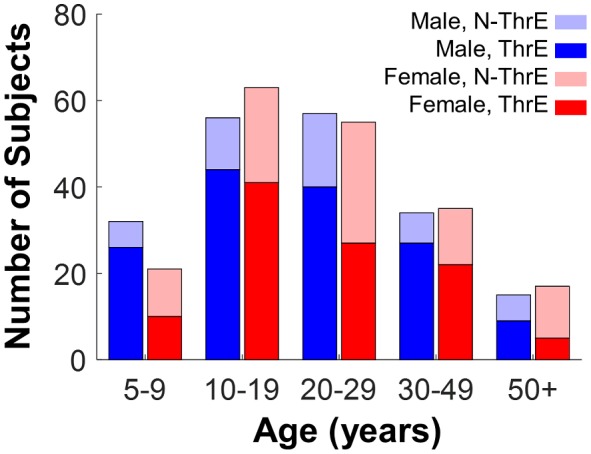
Demographics of subjects. Blue and red bars represent the number of male and female subjects. Dark blue and dark red indicate subjects who had throwing experience in each age bin. Light blue and light red bars represent the number of male and female subjects who had no throwing experience in each age bin.

The subject recruitment resulted in a well-balanced composition of gender across the age range. Figure 1 shows the number of subjects stratified into five age groups: 5–9 years: *n* = 53, 22 female; 10–19 years: *n* = 119, 68 female; 20–29 years: *n* = 112, 59 female; 30–49 years: *n* = 69, 40 female; 50+ years: *n* = 32, 21 female. Similar age groups have been used in other large-scale studies on motor skills (for example McKay et al., 2017). Before adopting these age categories, different age bins had been considered and the alternative stratifications did not change the main findings. The subject distribution shows a relatively even gender composition, even though no explicit attempts were made to achieve an equal number of males and females.

Although subjects were asked to report the number of years of throwing experience together with specific ball sports, they were only divided into two categories for analysis: “throwing experience” and “no throwing experience.” The more detailed responses were not analyzed further as they were very heterogeneous: the reported number of years was often left blank or ambiguous (e.g., “high school” or “when younger”) and many specific ball sports did not have a large enough sample size. Figure 1 and Table 1 differentiates the number of subjects with and without throwing experience. Note that males tended to have more throwing experience, as frequently reported in the literature.

**Table 1:** Percentage of male and female subjects who reported experience with ball throwing.

Age (years)	5–9	10–19	20–29	30–49	50+
Male	81.25%	78.57%	70.18%	79.41%	60.00%
Female	47.62%	65.08%	49.09%	62.86%	31.25%

As exclusion based on handedness was not permitted by the museum, participants were both right- and left-handed. In line with general population estimates, 46 (11.03%) subjects identified themselves as left-handed (Porac, 2016). For left-handers, the display was re-positioned to present the same task in mirror-reflected fashion to ensure the same challenge. However, many of the left-handed subjects reported being ambidextrous or preferred throwing with their non-dominant hand. After being given the choice to perform the task with their preferred hand, 24 (52.17%) of the subjects threw with their right hand, while 22 (47.83%) threw with their left hand. Finally, the subject pool drew from a variety of races and ethnicities that approximate the demographics of the U.S., even though there was a slightly higher representation of Asians and a lower representation of Black or African Americans (Table 2; Humes et al., 2011). Thus, our data and results represented a good cross-section of the population.

**Table 2:** Demographic distribution of participants.

Race	Number of participants	U.S. Demographics
Native Americans and	1 (0.24%)	0.90%
Alaska Natives
Asian	44 (10.55%)	4.80%
Black or African American	10 (2.40%)	12.60%
Native Hawaiian and Other	0 (0.00%)	0.20%
Pacific Islanders
White	337 (80.82%)	72.40%
Two or More Races	8 (1.92%)	2.90%
Prefer Not to Answer	17 (4.08%)	6.20%
or Some Other Race		

### Experimental Set-Up

**Figure 2:**
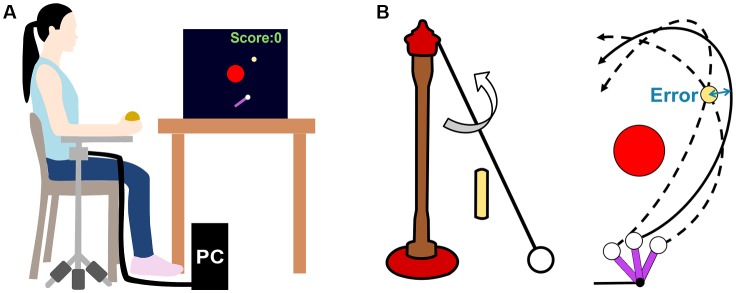
**(A)** Experimental set-up. Subjects are seated in front of the computer screen and grasp the ball affixed to the end of a lever arm, mounted on a tripod. **(B)** Left: Real skittles task: A ball attached to a vertical pole with a string. Right: Top-down projection of the skittles task presented on the computer screen. The purple bar rotates as subjects move their forearm about the elbow on the lever arm. The white circle representing the ball was released from the purple bar as the force sensor on the ball was released. Upon release, the elliptical trajectory of the ball’s path was shown. The two dashed trajectories show two different ball releases that resulted in a target hit; the solid trajectory shows a ball release that resulted in a non-zero performance error.

All participants played the virtual throwing game, while sitting in front of a computer screen (Figure 2A). To throw the ball, subjects placed their dominant forearm on a horizontal lever arm that allowed for a single-joint flexion and extension about the elbow. In contrast with previous studies using this set-up, a portable version was developed for easy assembly and storage at the Museum of Science. The lever arm was mounted on a tripod, with weights (9.0 kg) attached to each leg of the tripod for stability. Subjects were seated during this task and the tripod height was adjusted according to each subject’s arm height to ensure comfortable shoulder and arm position. The throwing task was modeled after the American playground game tetherball or the British pub game skittles, in which players throw a ball tethered to a post to hit a target (Figure 2B, left panel). In the experimental task, subjects threw the ball in a virtual environment to hit a target located on the far side without hitting the post (Figure 2B, right panel). The same task was used previously in laboratory experiments where participants stood in front of a back-projection screen (Cohen and Sternad, 2012; Zhang et al., 2018).

A 28-inch computer monitor displayed a top-down view of the throwing game. Subjects saw the red post (radius = 1.88 cm) centered at the origin and the yellow target (radius = 0.38 cm) located 3.23 cm above and 2.85 cm to the right of the center of the post. For subjects who threw the ball with their left hand, this target location was mirrored to 3.23 cm above and 2.85 cm to the left of the center post. The axle of the purple virtual lever arm (length = 0.3 cm) was located 11.25 cm below the origin, and the virtual white ball (radius = 0.03 cm) was attached to the distal end of the lever arm (Figure 2B, right panel). These coordinates were identical to the ones used in previous laboratory studies, only downscaled by 7.5% to fit on the monitor (e.g., Zhang et al., 2018).

Subjects were instructed to throw the ball such that it traveled through the center of the yellow target without hitting the red post. The error was calculated as the minimum distance between the center of the target and the ball trajectory (Figure 2B, right panel). When the ball trajectory went through the target, its color changed from yellow to green to signal a successful hit. While zero error was defined when the ball trajectory went through the center of the target, a threshold granted some tolerance for success: color change was implemented when the error was smaller than 0.38 cm on the screen (5 cm in workspace coordinates).

At the start of each throw, subjects grasped a wooden ball affixed to the distal end of the real lever arm and pressed their index finger on an analog force sensor (Interlink Electronics, Camarillo, CA, USA). Pressing the sensor attached the virtual ball to the end of the real lever arm on the monitor. Subjects then extended their arm and simultaneously released the ball by extending their index finger from the force sensor (similar to throwing a Frisbee). Right-handed subjects were instructed to throw from left to right, and left-handed subjects from right to left; the display was horizontally mirrored to maintain the same challenge. The lever arm’s movement was recorded using a digital encoder (BEI Sensors, Goleta, CA, USA) and was updated on the screen in real-time. Upon ball release, subjects saw the ball’s elliptical path on the screen for 1.4 s in real time. The ball’s trajectory was fully determined by the angular position and velocity of the lever arm at the moment of release, as defined by the task model.

### Experimental Procedure and Design

Before participating, each subject observed a previous subject or the experimenter play the virtual throwing game. Subsequently, he/she completed 10 practice throws before the data acquisition began. In the experiment, each subject threw the ball in 4 blocks of 25 throws each, for a total of 100 throws. A performance score was shown to subjects at the end of each block (Figure 2A). This score combined two contributions: achieving a target hit added 1 point, while hitting the post decreased the number by 1 point. Note that this entire testing session lasted maximally 15 min per museum policy. It is also important to keep in mind that even though the experiment was conducted in a corner of the Hall of Life, there was a lively and bustling atmosphere. Typically, children were curiously watching their siblings, and parents and relatives were waiting or in conversations with the other experimenters. Hence, compared to a laboratory study, the setting for this data collection was very uncontrolled.

### Task Model

A two-dimensional model was used to calculate the ball trajectory in the work space. In this model, the ball was attached by two orthogonal, massless springs to the origin, which was defined at the location of the post. Thus, its *x-* and *y-*positions could be computed for each time *t* using the following equations:

$$x(t)=A_{x}sin(\omega t+\varphi_{x})e^{-\frac{t}{\tau }}$$

$$y(t)=A_{y}sin(\omega t+\varphi_{y})e^{-\frac{t}{\tau }}$$

The frequency ω denotes the system’s natural frequency. The exponential term with relaxation time τ was included as a damping coefficient to approximate realistic behavior of the ball trajectory; it was set to 20 s. The amplitudes *A*_x_ and *A*_y_ and phases *φ*_x_ and *φ*_y_ were calculated from the angular position and velocity of the ball as determined by the recorded movement of the lever arm. As the restoring forces are proportional to the distance of the ball from the origin, the ball was accelerated toward the origin upon release (for details, see Hasson et al., 2016).

### Dependent Measures

During the experiment, the subject received feedback in the form of a running *score* displayed on the top right corner for the screen (Figure 2A). This score was increased by one point if the throw resulted in a target hit and decreased by one point if the ball hit the post. After each block, this score was displayed in the center of the screen; subsequently, the counting restarted for the next block. Hence, the maximum number of points for each block was 25.

To obtain a finer resolution of the accuracy of their throws, the *performance error* for each throw was calculated as the minimum distance between the ball’s path and the center of the target (Figure 2B). Throws that hit the center post had to be eliminated prior to further analysis because errors could not be calculated from these throws. To keep track of these failed throws, the number of post hits was summarized separately.

**Figure 3:**
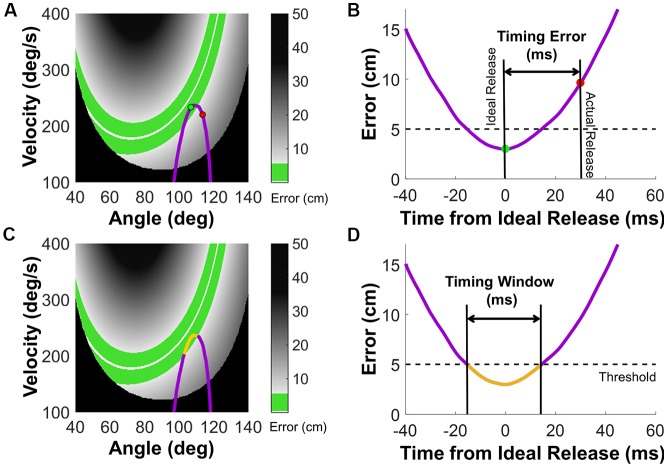
Calculation of timing error and timing window. **(A)** Visual representation of timing error in an exemplary throw. The result space spanned by angle and velocity at ball release is color-coded, with black representing post hits, and shades of gray indicating an error larger than 5 cm. The white line is the solution manifold, representing zero error, or balls whose trajectory went exactly through the center of the target. The green band around the solution manifold represents throws with errors less than the 5 cm threshold, which were also counted as target hits. The arm trajectory is shown in purple, and the subject’s release time is represented by the red point. The ideal release time is marked by the green point, representing the ball release that would have resulted in minimum error. **(B)** The purple trajectory depicts the performance error that would result from each point on the arm trajectory. The time was aligned with the time at which the minimum performance error would occur (green point). The timing error was the time difference between actual (red point) and ideal release (green point). Note that while the units of this measure are milliseconds, this is a spatiotemporal metric. **(C)** Visual representation of timing window with an exemplary throw. The arm trajectory is shown in purple. The yellow region along the arm trajectory marks the segment of the trajectory in which any release would result in a target hit. **(D)** The performance error against the time depicted in the same way as timing error. Timing window (yellow region) is the segment of the trajectory that would result in errors below the 5 cm threshold.

In addition to these outcome variables, two measures that quantified the process or execution were calculated. *Timing error* and *timing window* evaluated the timing of ball release that was central to an accurate target hit. These two metrics were calculated for each arm trajectory, using the model of the task and its result space for reference. As mentioned above, the task of throwing to a target is redundant as many, mathematically infinitely many, strategies can achieve a target hit. Both angle and velocity of the arm at ball release determine the ball trajectory and its error, and different combinations of release angle and release velocity can achieve the same error value. Figures 3A,C show the result space for the specific target constellation of this study. The set of all possible pairs of release angle and release velocity that achieve zero error are shown by the white line that defines the solution manifold. The green band around the solution manifold represents those releases that achieved a successful hit within the error threshold that was indicated by a color change of the target. The different gray shades represent different error magnitude; the black areas represent those throws that resulted in a post hit.

Based on this representation, *timing error* and *timing window* was calculated for each throw (Cohen and Sternad, 2012; Zhang et al., 2018). Figures 3A,C show an exemplary arm trajectory plotted in this result space (which is also state space purple curve). In Figure 3A, the green point indicates the ideal ball release leading to the lowest possible error for this trajectory; the red point indicates the actual ball release later in the trajectory that results in a higher error. To calculate the temporal difference between the ideal and actual release, the recorded arm trajectory was first converted from a sequence of angle-velocity pairs to an “error trajectory”: at each time point, the angle-velocity in the arm trajectory was regarded a ball release for which the task error was calculated (Figures 3B,D). This error trajectory was calculated with a resolution of 1 ms. The ideal release time is the minimum in this representation centered at zero; the actual time is shown by the red dot and the absolute difference defines the *timing error* (Figure 3B).

The *timing window* of each throw was defined as the amount of time that the error trajectory was within the success threshold of 5 cm. Figure 3C illustrates this in the same arm trajectory as above; the orange segment of the trajectory is within the green band indicating that ball releases lead to successful target hits. Figure 3D illustrates the calculations based on the error trajectory. A longer timing window presented a longer opportunity during which the subject could release the ball and hit the target, i.e., the timing of release did not have to be as accurate. Note that a longer timing window was not necessarily associated with a smaller timing error. Performance could improve independently by exploiting timing window or reducing timing error. Previous results showed that in this task, subjects first reduced timing error followed by a gradual increase of the timing window with extended practice (Zhang et al., 2018).

**Figure 4:**
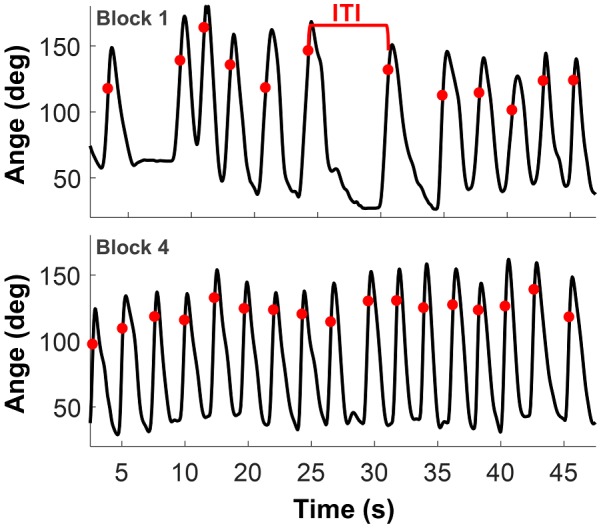
Arm trajectories and inter-throw intervals (ITI). The upper and lower panels show the angle of the lever arm during Block 1 and Block 4 from one subject. The red points mark the moments of ball release. The inter-throw interval was defined as the time between two successive ball releases.

To quantify an additional aspect of timing that is frequently discussed, *rhythmicity*, this aspect was included in the current analysis. While the throwing task itself did not require any rhythmicity, a previous study showed that people spontaneously fell into a rhythmic pattern when performing a long series of discrete throws (Zhang and Sternad, 2019). Rather than performing the individual throws separated by a pause of potentially irregular length, subjects tended to connect consecutive throws into a continuous rhythmic pattern. Figure 4 shows two exemplary time series of the arm trajectory, one early and one later in the session. As can be seen, the pauses between throws disappeared and the ball releases began to occur at approximately periodic intervals. This tendency to develop a rhythm over a sequence of similar actions was viewed as a manifestation of an individual’s sense of rhythm. Therefore, this study also measured this emerging rhythmicity.

To quantify the development of rhythmicity, the inter-throw-intervals (*ITI*) were calculated. To track the rhythmicity as it developed with practice in each subject, the variability of the *ITI*s was calculated in a moving window of 25 throws. Starting with the first throw, the windows were centered from the 13th throw to the 88th throw. As the distributions significantly deviated from normality, the quartile variation coefficient (*QVC*) was calculated:

$$QVC_{\text{ITI}}=\;\frac{Q3_{\text{ITI}}-Q1_{\text{ITI}}}{Q3_{\text{ITI}}+Q1_{\text{ITI}}}$$

where *Q1* and *Q3* refer to the 25th and 75th percentile of the distribution of *ITI*, respectively. The *QVC*_ITI_ of the last window of the trial sequence (centered on trial 88) was used to examine age and gender differences.

### Statistical Analysis

A first inspection of the data examined whether the relatively short practice time of 100 trials led to performance improvements. The trial means across subjects of the dependent measures, performance error, timing error and timing window, were fit with a power regression, separated by gender: *f*(*x*) = *ax*^b^, where *x* is the trial index. The parameters *a* and *b* were determined as those that minimized the sum of squares of *f*(*x*) between the fit and the dependent measure. A nonlinear least-squares solver was applied using a trust-region estimation algorithm (Matlab function “lsqnonlin”). The rhythmicity metric *QVC*_ITI_ was fit with the same power regression across 76 points (centers of the 25-point windows). The power function fits were compared with linear and exponential regressions, but their *r*^2^-values were considerably lower. This was likely due to fast initial familiarization with the set-up that was followed by relatively slow or negligible changes.

Following the inspection of the sequence of 100 trials, the dependent measures performance error, timing error and timing window in the final block were deemed best to represent the performance. Examining learning over all four blocks appeared too short and confounded. First, the practice time of 10 min is very short compared to regular laboratory experiments on motor learning that last 40–60 min, often repeated over several days. Second, the initial improvements seen in Figures 6–8 were largely due to familiarization and also focusing attention to the task, as the subjects participated as a break from their museum visit. Therefore, the final block was regarded best to reflect the subjects’ skill level before they would hone their skill with more extensive practice. The last block also showed the least variance, i.e., was best represented by the mean. The full data set of all 100 trials is shown as a time series in panels A below.

**Figure 5:**
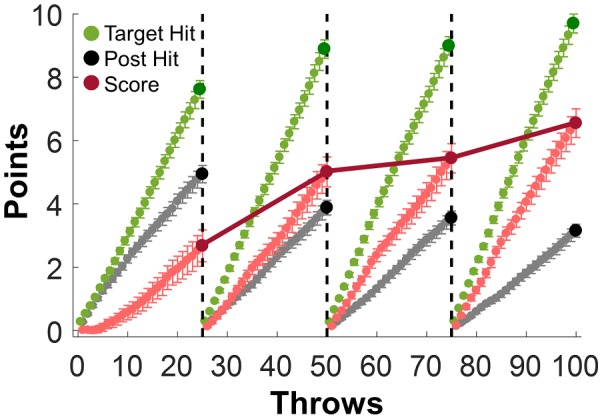
Number of target hits, post hits, and overall scores over practice. The green, black and red points represent the cumulative numbers of target hits, post hits and scores within each block, respectively. The dark red line highlights the final score of each block that was presented to the subjects as feedback.

**Figure 6:**
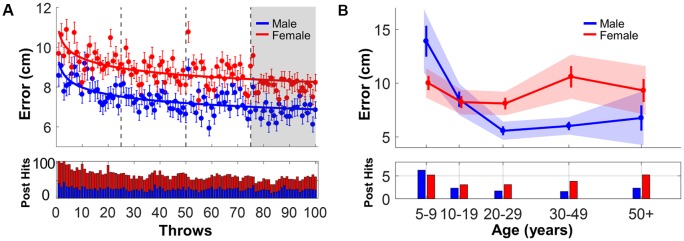
Performance error and post hits. **(A)** Upper panel: mean performance errors of male (blue) and female (red) subjects across 100 practice throws. Error bars represent the standard error across subjects for each trial. The red and blue curves are power functions regressed to the data. Lower panel: total number of post hits of each throw separated by gender. **(B)** Upper panel: mean performance error in the last block separated by age groups. Error bars represent standard errors. Shaded areas are the 95% confidence intervals. Lower panel: average number of post hits per gender and age bin in the last block.

**Figure 7:**
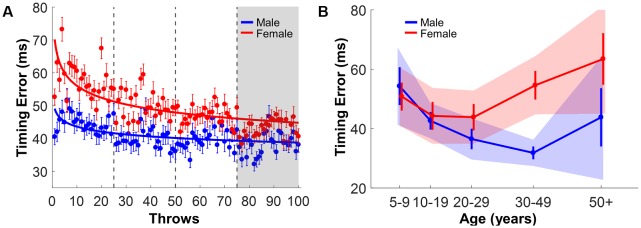
Timing error. **(A)** Timing error of male and female subjects across 100 practice throws. Error bars represent the standard error across subjects for each trial. The solid lines are power functions regressed to the data. **(B)** Mean timing error of the last block separated by gender and age group; error bars are the standard errors. The shaded areas are the 95% confidence intervals.

**Figure 8:**
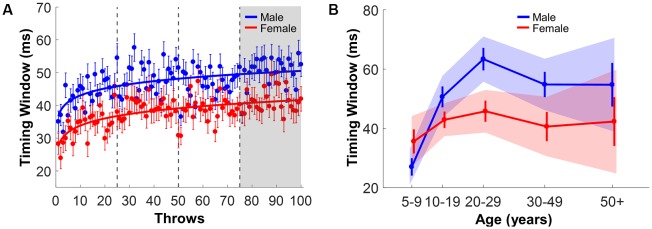
Timing window. **(A)** Timing window of male and female subjects across 100 practice throws; the error bars represent the standard error across subjects per trial. **(B)** Timing window of the last block separated by gender and age group; error bars are the standard errors. The shaded areas are the 95% confidence intervals.

Hence, to test the hypotheses, the mean values of the last 25 throws (block 4) were submitted to separate 2 (gender) × 5 (age group) × 2 (throwing experience) three-way analysis of variances (ANOVAs). The factors gender and throwing experience only had two categorial levels. To test the effect of age, participants were parsed into five age groups with different sample numbers: 5–9 years (*n* = 53), 10–19 years (*n* = 119), 20–29 years (*n* = 112), 30–49 years (*n* = 69), and 50+ years (*n* = 32). For exploration of the significant interactions, pairwise posthoc comparisons with Bonferroni adjustments were applied. The significance threshold α was set to 0.05. For the rhythmicity metric *QVC*_ITI_, the same ANOVA was run using the final window of 25 trials centered on trial 88.

When testing the distribution of the residuals of the four dependent variables for normality, it was revealed that the residuals for all variables did not pass the criteria for normality (Kolmogorov-Smirnov and Shapiro-Wilk tests). Using histograms and Q-Q plots, we found that the dependent variables were slightly skewed and lepto- or platykurtic. Therefore, we assessed the validity of the ANOVA results by additional tests using the non-parametric Kruskal-Wallis test for the main effects and posthoc pairwise comparisons. The results of these tests were consistent with the results from the ANOVAs. Note that numerous studies have reported robustness of the F-statistic with non-normal data, especially when the sample size is large as in our study (Donaldson, 1968; Ramseyer and Tcheng, 1973; Bevan et al., 1974). When reporting significance values of factors and interactions, partial eta-squared was reported as the effect size. For the multiple pairwise comparisons, Bonferroni corrections were applied. The effect sizes for significant posthoc results were indicated by Cohen’s *d*.

For all dependent measures, outliers were removed before calculating the mean or the window estimate for each subject: within each subject, trials in the last block that were outside three standard deviations of the mean were excluded. 4.3% of the entire trials were eliminated due to this criterion and none of the subjects were removed.

## Results

### Scores

Subjects received running feedback about their task performance in the form of a numeric score. This number combined two separate contributions: successful target hits (+1 point), and post hits (−1 point); the maximum score for each block with all target hits and no post hits would be 25 points. Figure 5 shows the participant averages of the score and the two separate metrics post hits and target hits across the four blocks; the displayed scores were reset after each block. All values increased within each block. More importantly, the final scores of each block increased: Block 1: 2.68, Block 2: 5.01, Block 3: 5.44, Block 4: 6.55 points. This first overview result demonstrates that subjects were indeed able to improve their task performance within the short practice duration. Nevertheless, the task remained challenging as the scores remained far from the maximum of 25 points at the end of the experimental session.

### Performance Error

A more fine-grained metric to quantify throwing accuracy was the distance between the ball trajectory and the target for each throw. Figure 6A depicts how the performance error decreased over the 100 throws. The points represent the mean performance error separated for male or female subjects for each throw; the points are means of the male and female subjects and the error bars indicate standard errors across throws. The mean errors were fitted with power functions separately for each gender; the *r*^2^-values were 0.46 and 0.42 for male and females, respectively. Table 3 lists the fitting parameters and their confidence intervals. The parameter *a* quantifies the level, while the parameter *b* quantifies the change. The asterisks indicate that the *a*-parameter was outside the 95% confidence band of the other gender’s fit, while the *b*-parameters did not differ. This underscores that male subjects showed overall better performance than females, while the rate of change did not differ. These results contradicted *Hypothesis 1*. The bottom panel of Figure 6A shows the declining number of post hits over practice, split by gender. Consistent with the error results, the histograms showed that females had more post hits than males considering the number of subjects in each gender were approximately equal (191 females and 194 males); hence, this exclusion of trials did not confound but rather support the difference seen in the error results.

**Table 3:** Parameters and *r*^2^-values of the power regressions.

Dependent measures	Male			Female		
	*a*	*b*	*r*^2^	*a*	*b*	*r*^2^
Performance	9.26*	−0.06	0.46	10.81*	−0.06	0.42
Error	(8.80, 9.72)	(−0.08, −0.05)		(10.29, 11.34)	(−0.07, −0.05)
Timing	49.08*	−0.05*	0.28	70.15*	−0.10*	0.55
Error	(46.17, 51.99)	(−0.07, 0.04)		(65.88, 74.42)	(−0.11, −0.08)
Timing	36.70*	0.07	0.32	27.54*	0.09	0.44
Window	(33.71, 39.69)	(0.05, 0.09)		(25.21, 29.87)	(0.07, 0.11)
Rhythmicity	0.10*	−0.08*	0.95	0.12*	−0.10*	0.95
(*QVC-ITI*)	(0.10, 0.11)	(−0.09, −0.08)		(0.12, 0.12)	(−0.11, −0.10)	

*The values in parentheses are the boundaries of the 95% confidence interval. The asterisks indicate where the parameters of each gender are outside the 95% confidence interval of the other gender. Note the *r*^2^-values for the rhythmicity measure are inflated because the *QVC-ITI* values were calculated with overlapping windows*.

The three-way ANOVA on performance error revealed that the Gender × Age interaction was significant: *F*_(4,365)_ = 4.65, *p* = 0.001, *η*^2^ = 0.05, together with a significant main effect of Age, *F*_(4,365)_ = 9.19, *p* < 0.001, *η*^2^ = 0.09, and Gender, *F*_(1,365)_ = 7.99, *p* = 0.005, *η*^2^ = 0.02 (Figure 6B). Pairwise posthoc tests aimed to identify age groups that show gender differences (one comparison per age group). The results showed that differences in performance error between male and female subjects did not arise until after the age of 20 years, i.e., with the third age group. Male subjects had significantly lower errors than female subjects in the age bracket of 20–29, *t*_(110)_ = −3.56, *p* = 0.005, *d* = 0.63, and for ages 30–49, *t*_(67)_ = −4.76, *p* < 0.001, *d* = 1.0. However, this gender difference disappeared for individuals older than 50 years (Figure 6B). Thus, contrary to *Hypothesis 1*, there was a difference in performance between male and female subjects in the middle-aged subjects. The bottom panel of Figure 6B shows the average post hits during Block 4 for each age and gender group.

In addition, the Gender × Throwing Experience interaction was weakly significant, *F*_(1,365)_ = 4.00, *p* = 0.04, *η*^2^ = 0.01. Using posthoc tests, all possible combinations at the level of Gender × Throwing Experience were compared (six comparisons). Amongst those individuals without throwing experience, the male subjects were significantly more accurate than females, *t*_(132)_ = −3.48, *p* = 0.005, *d* = 0.61. However, this gender difference disappeared between the subgroups with throwing experience. This result suggests that while females may be disadvantaged amongst “novices,” females can catch up in throwing skill with practice.

### Timing Error

Like performance error, Figure 7A shows that timing error declined and stabilized over practice. The mean values of male and female subjects were fit with two power functions that achieved *r*^2^-values of 0.28 (males) and 0.55 (females). Both *a-* and *b*-parameters of males and females were outside the confidence intervals of the other gender, underscoring the visible difference between the two power functions (Table 3). As for performance error, this result rejected *Hypothesis 1*, and was consistent with the alternative hypothesis. To examine the effect of age and experience, the performance means of the last 25 throws were submitted to the same three-way ANOVA. A significant Gender × Age interaction was found, *F*_(4,365)_ = 2.56, *p* = 0.04, *η*^2^ = 0.03, together with significant main effects of Gender, *F*_(1,365)_ = 9.77, *p* = 0.002, *η*^2^ = 0.03, and Age, *F*_(4,365)_ = 2.69, *p* = 0.03, *η*^2^ = 0.03 (Figure 7B). Pairwise posthoc comparisons between males and females across age groups (five comparisons) revealed that females made more timing errors than males, but only in the 30–49 years age group, *t*_(67)_ = −4.26, *p* < 0.001, *d* = 0.92.

### Timing Window

Figure 8A shows the timing window increasing as practice progresses. The power functions on the mean values for male and female subjects again highlighted longer timing windows in male subjects across all trials (*r*^2^ for males: 0.32, *r*^2^ for females: 0.44). These overall elevated values for males are reflected in the different *a*-parameters (Table 3). However, the change parameters *b* were within the other gender’s confidence interval, indicating a similar time course over the 100 trials. This result is consistent with the two other metrics, rejecting *Hypothesis 1* and favoring the alternative hypothesis.

Submitting the mean timing windows of block 4 to the same three-way ANOVA, several significant interactions emerged: Gender × Age, *F*_(4,365)_ = 2.51, *p* = 0.04, *η*^2^ = 0.03, Age × Throwing Experience, *F*_(4,365)_ = 3.18, *p* = 0.01, *η*^2^ = 0.03, and Gender × Throwing Experience, *F*_(1,365)_ = 5.54, *p* = 0.02, *η*^2^ = 0.02. The main effects of Age, *F*_(4,365)_ = 6.63, *p* < 0.001, *η*^2^ = 0.07, and of Gender, *F*_(1,365)_ = 6.50, *p* = 0.01, *η*^2^ = 0.02, were also significant (Figure 8B). Pairwise comparisons of Gender across age groups (five comparisons) revealed that male subjects achieved significantly higher timing windows than female subjects, but only in the 20–29 age group, *t*_(110)_ = 3.08, *p* = 0.015, *d* = 0.56. All possible combinations of Gender and Throwing Experience were tested using six posthoc comparisons. The results indicated that when subjects reported no throwing experience, females lagged males in these metrics, *t*_(132)_ = 3.19, *p* = 0.01, *d* = 0.55. However, this difference leveled out for individuals that reported throwing experience. Consistent with the results for performance error, the gender difference of timing window selectively manifested in young adults and those who had no throwing experience.

### Rhythmicity

**Figure 9:**
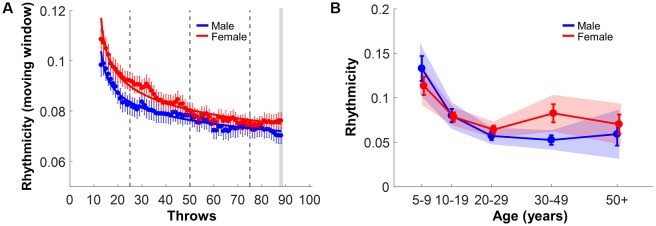
Rhythmicity. **(A)** Rhythmicity measured by quartile variation coefficient (QVC)-inter-throw-intervals (ITI) in a moving window of 25 trials across practice throws in both males and females; error bars represent the standard error across subjects per window. **(B)** *QVC-ITI* of the last block (corresponding to the last two points in panel **A**) separated by gender and age group; error bars represent standard errors. The shaded areas are the 95% confidence intervals.

Variability of the inter-throw interval *QVC*_ITI_ was examined applying a moving window of 25 throws centered from the 13th throw to the 88th throw. Figure 9A shows how *QVC*_ITI_ decreased with practice, demonstrating that the movements became more rhythmic across the short practice. The same power function fits were applied on the subject means across each trial (Table 3). The initial estimates of *QVC*_ITI_ started with 0.10 and 0.11 and declined to 0.07 and 0.08 for males and females, respectively. As the overlapping windows significantly smoothed the time course, the function fits had expectedly higher *r*^2^-values of 0.95 with much tighter confidence intervals. Therefore, it was not surprising that the confidence intervals of the *a-* and *b*-parameters did not overlap, suggesting higher variability or less rhythmicity in female participants, counter *Hypothesis 2*.

However, this trend was not replicated when the last timing window (centered on the 88th throw) was submitted to the ANOVA (Figure 9B). A significant main effect of Age, *F*_(1,365)_ = 12.71, *p* < 0.001, *η*^2^ = 0.12, and subsequent posthoc tests for all combinations between the age groups (10 comparisons) revealed higher variability in the younger participants (5–9 years) compared to all other age groups (*p* < 0.001). There were no significant effects involving gender, suggesting the rhythmic timing ability did not differ in male and female subjects, consistent with *Hypothesis 2*.

## Discussion

Motivated by the recent call of the National Institute of Health for more inquiry into sex differences, this study re-visited the widely reported fact that male individuals outperform females in throwing and aiming. Numerous previous studies in the developmental literature examined sex differences and discussed the nature-nurture question by comparing cohorts of different socio-economic, educational and ethnic status, with or without experience or exposure to ball skills, and before and after training, typically focusing on early childhood to early adolescence (Thomas and French, 1985; Burton and Rogerson, 2003; Barnett et al., 2010; Gromeier et al., 2017). Specifically throwing revealed large differences between males and females. One limitation of most previous studies in this literature is that they used test batteries with rating scales or they relied on coarse-grained outcome measures such as movement time, errors or subjective appraisals of the “process” (Gromeier et al., 2017; McKay et al., 2017). To better understand gender-specific developmental processes, analyses must go beyond descriptive scores and quantify movement kinematics. Therefore, this study employed computational methods to detail kinematic features of a simplified throwing skill to further scrutinize this gender disparity across the lifespan.

With rare exceptions, the focus has been on overarm throwing, as exemplified in baseball and in pre-historic spear hunting. This whole-arm and whole-body action requires specific shoulder mobility, muscular strength and speed, the latter of which is enhanced by longer limb segments (Lombardo and Deaner, 2018a,b). All of these anatomical and physiological components are known to be more developed in age-matched males, especially after the onset of puberty. Hence, we conjectured that if throwing is modified to a sensorimotor skill eliminating the whole-arm and whole-body action and focusing on the coordination and timing of ball release, then the gender disparity should disappear.

When accurate targeting is the goal, subtle spatiotemporal coordination of the ball release emerging from the arm trajectory critically determines success. Returning to the developmental literature, behavioral tests of a broad range of tasks on spatiotemporal abilities revealed different gender-specific results (for a compact summary of sensorimotor differences in both sexes see Baker and Cornelson, 2018). While judging spatial relations, mental transformations and discrete time estimation have revealed a male advantage, rhythmic timing as in synchronizing to a metronome has not detected gender differences. Using a large cohort of all ages, we examined gender differences in a virtual task and evaluated not only task performance but also additional spatiotemporal aspects inherent to throwing.

As our throwing task was simplified to conform to a physical model that was implemented in a virtual environment, the task analysis allowed to quantify three separate metrics of coordination evaluated in state space: (1) the timing of ball release could be analyzed with respect to the optimal release defined by the solution manifold; (2) the spatiotemporal evolution of the arm trajectory could be evaluated with respect to the solution manifold to render a metric for the sensitivity of ball release to error and noise; and (3) the arm trajectory could be continuously recorded and revealed an emerging rhythmicity that could be quantified. These three metrics are independent of each other. While they all have the units of time, they characterize spatiotemporal aspects of coordination.

Prior to discussing the results, it is important to mention that our experiment differed from the many other cross-sectional studies on motor ability in several ways. Our recruitment relied on museum visitors that spontaneously volunteered out of curiosity to learn about and experience ongoing research. The recruiting procedure did not allow to select a specific number of subjects per age group or gender, nor to apply any other in/exclusion criteria, although the pool was confined to museum-goers. The data collection over 8 months resulted in more than 400 participants, whose age, gender and ethnic distribution were comparable to the U.S. demographics (Tables s 1, 2). The questionnaire had to be short as the entire experiment was limited by museum policy to 15 min. Participants performed the virtual task in a public place, often surrounded by other spectators and ongoing commentary by friends and relatives. Hence, this “field study” did not allow for the same undivided concentration as typical in laboratory studies. Nevertheless, the results on performance error were overall consistent with previous laboratory results and behavioral reports, hence proved remarkable robustness. While a sample of 400 participants is not very large for behavioral testing, our experimental station enabled quantitative data acquisition comparable to laboratory studies where 400 participants is an unprecedented large number.

### Performance Error

The most prominent result was that male subjects showed overall better performance across the 100 trials than females. However, this disparity only appeared in adult subjects, while younger subjects in the two age groups of 5–9 and 10–20 years performed similarly well. This result differed from the meta-analysis by Thomas and French (1985), where the mean effect sizes for age and gender for throwing velocity and distance throwing were already large in childhood and widened further during adolescence. This also differed from a previous study on the same virtual throwing task with a very small cohort of age 12–13 years that identified higher initial variability in females (Müller and Sternad, 2004a). In contrast, the comparable performance in the younger groups was consistent with *Hypothesis 1* that when throwing was stripped of its anatomical and physiological elements, the male advantage would disappear.

Why did the gender disparity emerge after age 20? To successfully execute this sensorimotor task, the participant needed to interpret the virtual objects, map the release angle and velocity to the performance error, transform arm movements into the 2-dimensional screen display and adjust arm movement according to the perceived error. This visuomotor processing may require cognitive abilities where adult males previously tended to show an advantage (Miller and Halpern, 2014; Wong, 2017). For example, males have performed better in tasks involving spatial perception and mental rotation, although not in spatial visualization (Voyer et al., 1995). However, these results were not as strong and unanimous as would be desired and many results remained descriptive with a lack of precise and principled boundaries between different tasks. A look into brain development is also problematic as it is hard to map any of the behavioral features onto specific brain areas. Further, the growth of cortical volume has a nonlinear time course and the change varies across areas and gender (Reiss et al., 1996; Lenroot et al., 2007). Therefore, it is premature to link the development of specific motor skills to changes in cortical volume. Much more work is needed to reveal the neural mechanisms underlying sex differences insd spatiotemporal abilities.

### Spatiotemporal Measures

In previous studies of motor skill, throwing was predominantly evaluated by qualitative scores (Barnett et al., 2010) and hitting errors (Thomas and French, 1985; Wong, 2017). The model-based virtual task of this study facilitated the detailed quantification of the arm trajectory and ball release in state space. Prior analysis of the entire result space permitted quantification of individual trajectories with reference to all possible solutions. Individual executions were characterized by two metrics: the timing of ball release and the shaping of the arm trajectory, referred to as timing error and timing window (even though the metrics captured spatiotemporal coordination). Previous laboratory studies on the same task showed that performance could be improved by two independent strategies: minimizing the timing error of the ball release and maximizing the error-tolerance of the arm trajectory by lengthening the timing window (Cohen and Sternad, 2012; Zhang et al., 2018).

All subjects in this study improved their timing error and timing window with practice, even though they only practiced 100 throws. Counter to *Hypothesis 1*, males outperformed females in both metrics. One difference was that female subjects decreased their timing error faster than males, while timing window evolved at the same rate for both genders. The fact that improvement in timing error was fast in early trials is consistent with previous adult results, showing that timing window was a more intricate feature that was optimized in later practice (Zhang et al., 2018). One note of caution to avoid overinterpretation of the results: the observable improvement in performance scores is likely to be ascribed to familiarization to the device, rather than learning that is defined by long-lasting effects indicative of neural changes. The 100 trials are too short to allow for a proper assessment of the ability to learn. Our typical laboratory studies comprise up to 2,000 trials or in different tasks up to 2 months of regular practice and reveal changes throughout the entire time course that also display long-lasting changes (Park et al., 2013; Park and Sternad, 2015; Huber et al., 2016). We, therefore, refrained from analyzing the time course of improvements any further and rather evaluated a snapshot of the performance in the last block.

### Experience in Throwing and Video Games

An important consideration for the interpretation of these results is exposure to ball throwing, i.e., the influence of environment and “nurture.” First, many more males than females reported previous involvement in ball sports across all ages (Table 2). However, this more extensive exposure was not simply associated with improved performance, and specifically in our data, males did not gain much from this experience. It was only females that showed a significant benefit from experience in ball sports and that annulled the gender differences present in those sub-groups without experience. This advantage from more practice speaks against the “nature” or the evolutionary argument, as for example proposed by Lombardo and Deaner (2018a,b). However, it needs to be kept in mind that this study examined a virtual aiming task that focuses on only one aspect of the whole-body throwing.

This dependence on experience was replicated in the spatiotemporal metrics of timing window, which shows a pronounced effect and suggests that this trajectory shaping was a subtle but robust estimate of throwing skill, as implicated in our previous study. Zhang et al. (2018) reported that in adults with 6 days of practice this shaping of the arm trajectory was a significant contribution to hitting success that increased in importance later in practice. The fact that this subtle feature was even measurable in this “field study” is noteworthy. While the discussion of throwing experience cannot be separated from performance, it should be kept in mind that the self-reports *via* questionnaires were not as accurate as would be desirable and responses had to be pooled into only two categories.

Another possible factor that may confound this gender attribution is experience in action video games that may have benefitted the interaction with the virtual environment. It has been argued that video gaming may train the ability of visual selective attention and spatial resolution of vision (Green and Bavelier, 2003, 2006, 2007; Castel et al., 2005). Action video games have been shown to be more appealing to boys than girls (Terlecki and Newcombe, 2005; Quaiser-Pohl et al., 2006); thus, boys may have developed better ability in spatial cognition due to their video game experience. Unfortunately, our questionnaire did not include a question on video gaming. That said, while a likely confound, the disparity between genders became only pronounced in the age group of 20–50 years, which included generations where video gaming may not be as prevalent as in the current youth. If video gaming was the predominant explanation for the gender differences, differences should have been seen at the younger age groups.

### Rhythmicity

A less obvious aspect of this discrete throwing task is the observed tendency to develop a rhythm when performing a series of similar movement. This anecdotal observation was quantified in a previous study using the same virtual task which showed that subjects spontaneously merged the discrete throws into a periodic and stable pattern (Zhang and Sternad, 2019). Similar to this previous study, the variability of the inter-throw intervals decreased from approximately 10% to 7% within only 100 throws. These values were even elevated because younger individuals had significantly higher variability; participants age 20 years and older reached 5%, comparable to previous reports. This level of consistency of an inter-throw-interval is similar to what is measured when subjects explicitly tap to a periodic metronome (Repp and Steinman, 2010; Repp, 2010).

Interestingly, the differences between genders were not as pronounced as in the two other spatiotemporal metrics. Even though the power functions tracing the evolution of rhythmicity over practice showed slightly better values for males, this difference was biased as overlapping timing windows narrowed the confidence intervals and facilitated the detection of a statistical difference. When only the final window was examined, gender differences disappeared across all age groups, consistent with *Hypothesis 2*. It needs to be emphasized that rhythmicity is independent from the timing error and window: timing error can be high even if the successive throws occur at exactly periodic intervals. One noteworthy fact was that younger subjects were considerably more variable, consistent with findings that rhythmic ability is developing in young children (Smoll, 1974; Volman and Geuze, 2000; Mastrokalou and Hatziharistos, 2007). In addition, lack of concentration and ease of distraction may have been an added factor that may have affected a consistent rhythm.

Previous fMRI studies documented that rhythmic movements are associated with fewer cortical and subcortical areas than discrete movements of the same limb (Schaal et al., 2004). It is tempting to speculate that the development of a rhythmic pattern may “economize” neural resources needed for executing a series of throws. Nevertheless, it is noteworthy to recognize that more rhythmic performance also tended to eliminate the pauses between throws. Eliminating pauses implies that the error processing between throws that is usually associated with performance improvements is time-constrained and has to occur while movement is ongoing. Therefore, the trend towards continuous rhythmic performance is counter-intuitive at first sight. The fact that both sexes showed a similar development underscored that rhythmicity is a fundamental human tendency, consistent with the notion of dynamic primitives (Strogatz and Stewart, 1993; Sternad, 2008; Hogan and Sternad, 2012, 2013; Zhang and Sternad, 2019).

### Conclusions

This large cross-sectional study on spatiotemporal coordination in throwing revealed that the widely reported male advantage in throwing may need some qualifications: previous behavioral testing invariably tested overarm throwing where anatomical and muscle physiological differences are the predominant contributors. This novel “field study” examined whether these differences remained when aiming was in focus and other physical factors were eliminated. However, performance scores and finer-grained measures of spatiotemporal coordination continued to show some male advantage. While overall sex differences remained across practice, the age-dependent analysis revealed that these only arose from age 20 years onwards and that in individuals with throwing practice, performance disparities leveled out. Rhythmicity was the only metric where gender did not show differences, speaking to a general human tendency to fall into rhythm. These results also highlight that more research on neural mechanisms is needed to unravel sex differences in sensorimotor control.

## Ethics Statement

All subjects gave written informed consent in accordance with the Declaration of Helsinki. The protocol was approved by the Institutional Review Boards of Northeastern University and the Museum of Science, Boston, MA, USA.

## Author Contributions

DS: design of the experiment. DC, ZZ and many more students: data collection. DC, ZZ and S-WP: data analysis and figure preparation. DC and DS: manuscript draft. DC, ZZ, S-WP and DS: interpretation of results and editing and approval of final manuscript.

## Conflict of Interest Statement

The authors declare that the research was conducted in the absence of any commercial or financial relationships that could be construed as a potential conflict of interest.
